# Supplementary material for: Development of an α-synuclein knockdown peptide and evaluation of its efficacy in Parkinson’s disease models
Source: Commun Biol. 2021 Feb 19;4:232. doi: 10.1038/s42003-021-01746-6 (PMC7895943; doi:10.1038/s42003-021-01746-6)
Supplement: Supplementary file 2 — Supplementary Information [file 42003_2021_1746_MOESM2_ESM.pdf]

Supplementary Figure 1

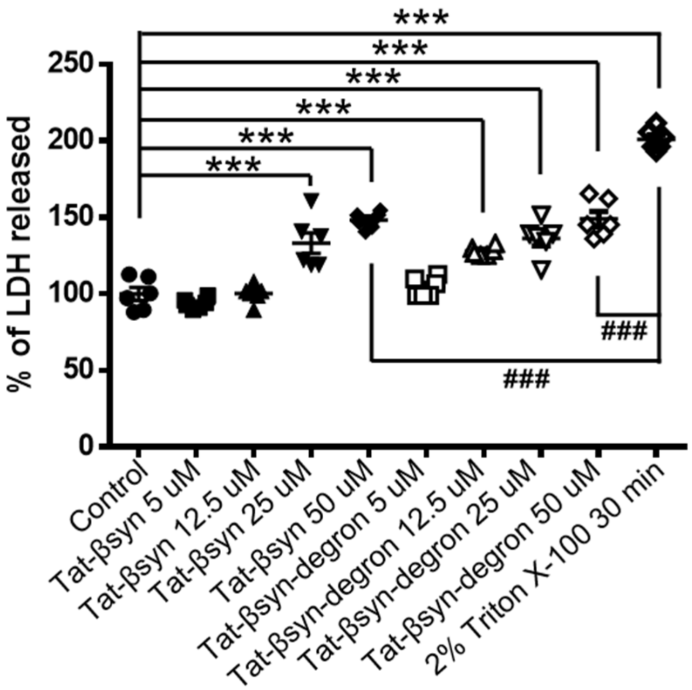

Supplementary Figure 1. Peptide cytotoxicity. DIV 14 primary cortical neurons were treated with different doses (5 µM, 12.5 µM, 25 µM, 50 µM) of the Tat-βsyn-degron and Tat-βsyn peptides and levels of released LDH in the culture medium were measured 24 hours after peptide treatment. The culture medium from cells treated with 2% Triton X-100 for 30 min at 37 °C was used as the positive assay control and the culture medium from untreated cells was used as the negative assay control. Both peptides induced similar and dose-dependent increase of LDH levels in the culture medium compared with the untreated control (N=6 for all the groups, $F(9,50) = 76.07$, $P<0.001$). Triton X-100 induced significantly higher LDH release levels than the peptide groups. Statistical significance was determined by one-way ANOVA, followed by Tukey's HSD *post hoc* test.

***P≤0.001 compared with the untreated control. ###P≤0.001 indicates differences between 50 uM peptide groups and the Triton X-100 group.

Supplementary Figure 2

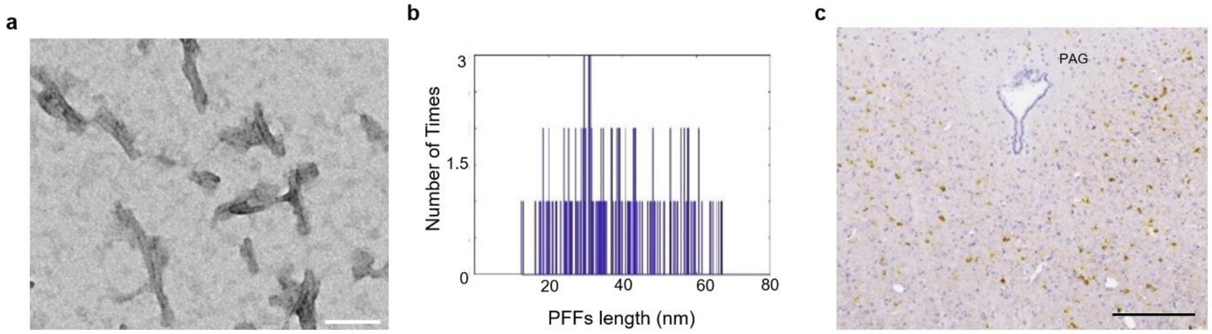

Supplementary Figure 2. Establishment of a mouse model of synucleinopathy. (**a**) Representative photomicrograph of PFFs by negative staining and visualized using Tenai 120v electron microscope. (**b**) Histogram showing the PFFs length distribution (length average= 40.68 nm, median length= 37.84 nm, minimal length = 12.78 nm, maximal length= 125.82 nm). (**c**) An example of immunohistochemical quantification in the periaqueductal grey (PAG) area showing phosphorylated p-S129 synuclein positive-areas surrounded in yellow. Scale bar: 100 nm in **a** and 200 µm in **c.**

Supplementary Figure 3

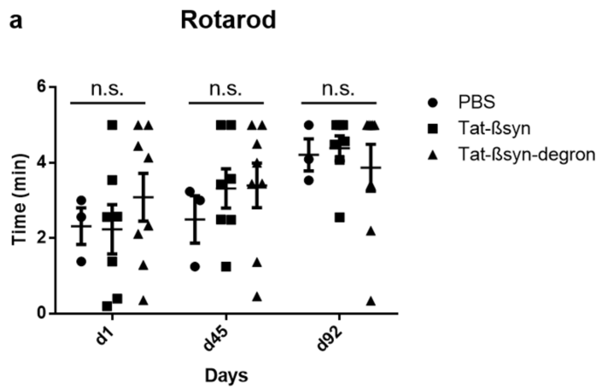

Supplementary Figure 3. Behavioral motor testing. (**a** and **b**) Histograms showing the results of the (**a**) rotarod and (**b**) wire-hang tests performed in M83 mice that received i.c. injections (PBS or PFFs) and i.p injections (PBS, Tat-βsyn or Tat-βsyn-degron). PBS-PBS group (N=3), PFFs- Tat-βsyn group (N=7), PFFs- Tat-βsyn-degron group (N=8). Motor function were not found to be different between any of the groups shown in (**a**) the rotarod test at day 1 (d1) ($F (2, 15) = 0.54$, $P= 0.591$), day 45 (d45) ($F (2, 15) = 0.43$, $P=0.660$) and day 92 (d92) ($F (2, 15) = 0.28$, $P=0.762$), or (**b**) the wire-hang test at d1 ($F (2, 15) = 1.29$, $P = 0.303$), d45 ($F (2, 15) = 0.20$, $P =0.821$) and d92 ($F (2, 15) = 0.90$, $P =0.427$). Statistical significance was determined by one-way ANOVA, followed by Bonferroni post hoc test. n.s. denotes not significant.

Supplementary Figure 4

β-synuclein [Homo sapiens], NCBI Accession: NP_003076.1, GI: 4507111
α-synuclein [Homo sapiens], NCBI Accession: P37840.1, GI: 586067

```
β-synuclein  1    MDVFMKGLSMAKEGVVAAAEKTKQGVTEAAEKTKEGVLYVGSKTREGVVQGVASVAEKTK  60
α-synuclein  1    MDVFMKGLSKAKEGVVAAAEKTKQGVAEAAGKTKEGVLYVGSKTKEGVVHGVATVAEKTK  60

β-synuclein  61   EQASHLGGAVFS-----------GAGNIAAATGLVKREEFPTDLKPEEVAQEAAEEPLIE  109
α-synuclein  61   EQVTNVGGAVVTGVTAVAQKTVEGAGSIAAATGFVKKDQLG---KNEE---GAPQEGILE  114

β-synuclein  110  PL-MEPEGESYEDPPQEEYQEYEPEA  134
α-synuclein  115  DMPVDPDNEAYEMPSEEGYQDYEPEA  140
```

Supplementary Figure 4. α-synuclein and β-synuclein are highly homologous. Homology alignment of α- and β-synuclein using NCBI's Protein BLAST shows that α- and β-synuclein are similar in protein sequences and that the amino acids 36-45 of β-synuclein (βsyn) used as the binding domain for α-synuclein (GVLYVGSKTR, highlighted in red and also underlined) is identical to the amino acid sequence 36-45 (GVLYVGSKTK) of α-synuclein, except for the last amino acid, which is R in β-synuclein, but K in α-synuclein.

Supplementary Figure 5

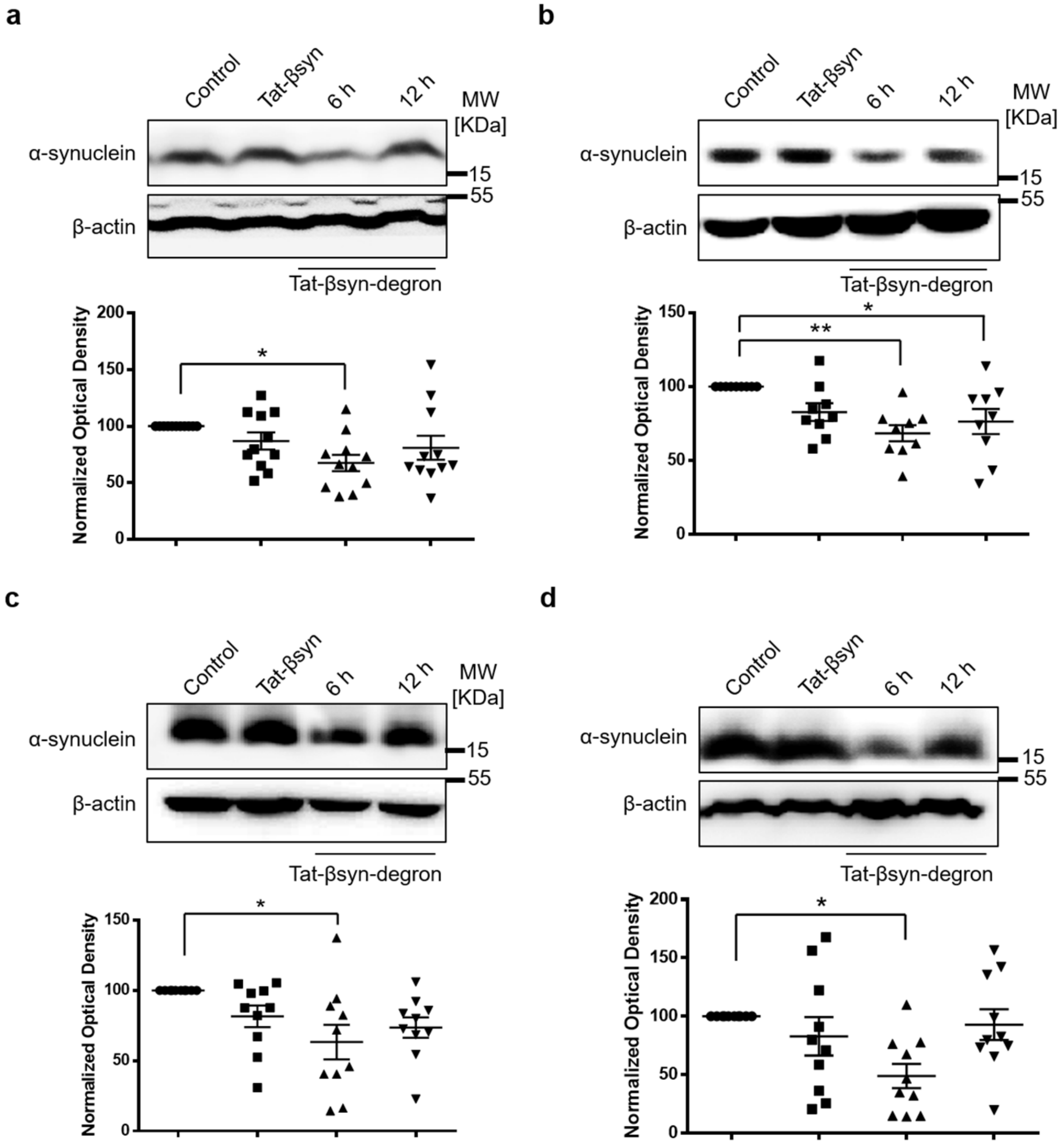

Supplementary Figure 5. Tat-βsyn-degron peptide knocks down endogenous α-synuclein in various tissues in C57BL/6 mice. C57BL/6 mice received a single dose of Tat-βsyn-degron or its control Tat-βsyn (6 μmol/kg; i.p.). (**a-d**) Immunoblots showing that acute treatment of Tat-βsyn-degron peptide induced significant decrease in the level

of endogenous α-synuclein in the kidney (**a**; N=11; $F(3,40)=3.26$; $P<0.05$), the spleen (**b**; N=9; $F(3,33)=5.68$; $P<0.01$), the striatum (**c**; N=10; $F(3,36)=3.68$; $P<0.05$) and the ventral midbrain (**d**; N=10; $F(3,36)=3.73$; $P<0.05$) at 6 hrs after injection, which gradually recovered around 12 hrs. In contrast, Tat-βsyn peptide had little effect on the α-synuclein level in any of the tissues at 6 hrs after injection (Bonferroni *post hoc* test, Tat-βsyn *vs* control: $P=1.00$ in the kidney; $P=0.23$ in the spleen; $P=0.70$ in the striatum; $P=1.00$ in the ventral midbrain). Data are presented as mean±S.E.M. The statistical difference between groups was determined by one-way ANOVA, followed by Bonferroni *post hoc* test. *$P\leq0.05$ and **$P\leq0.01$ compared with the control.

Supplementary Figure 6

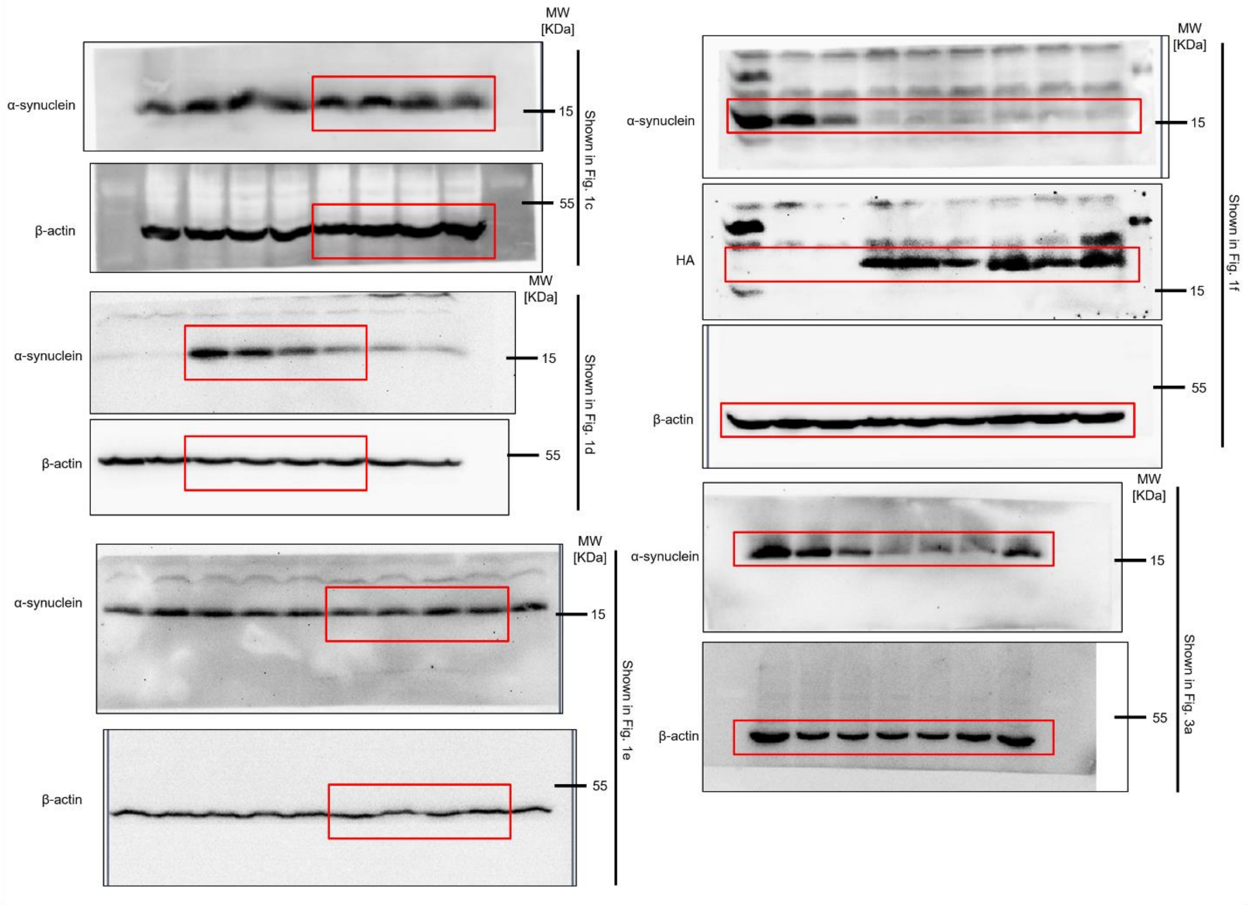

Supplementary Figure 6

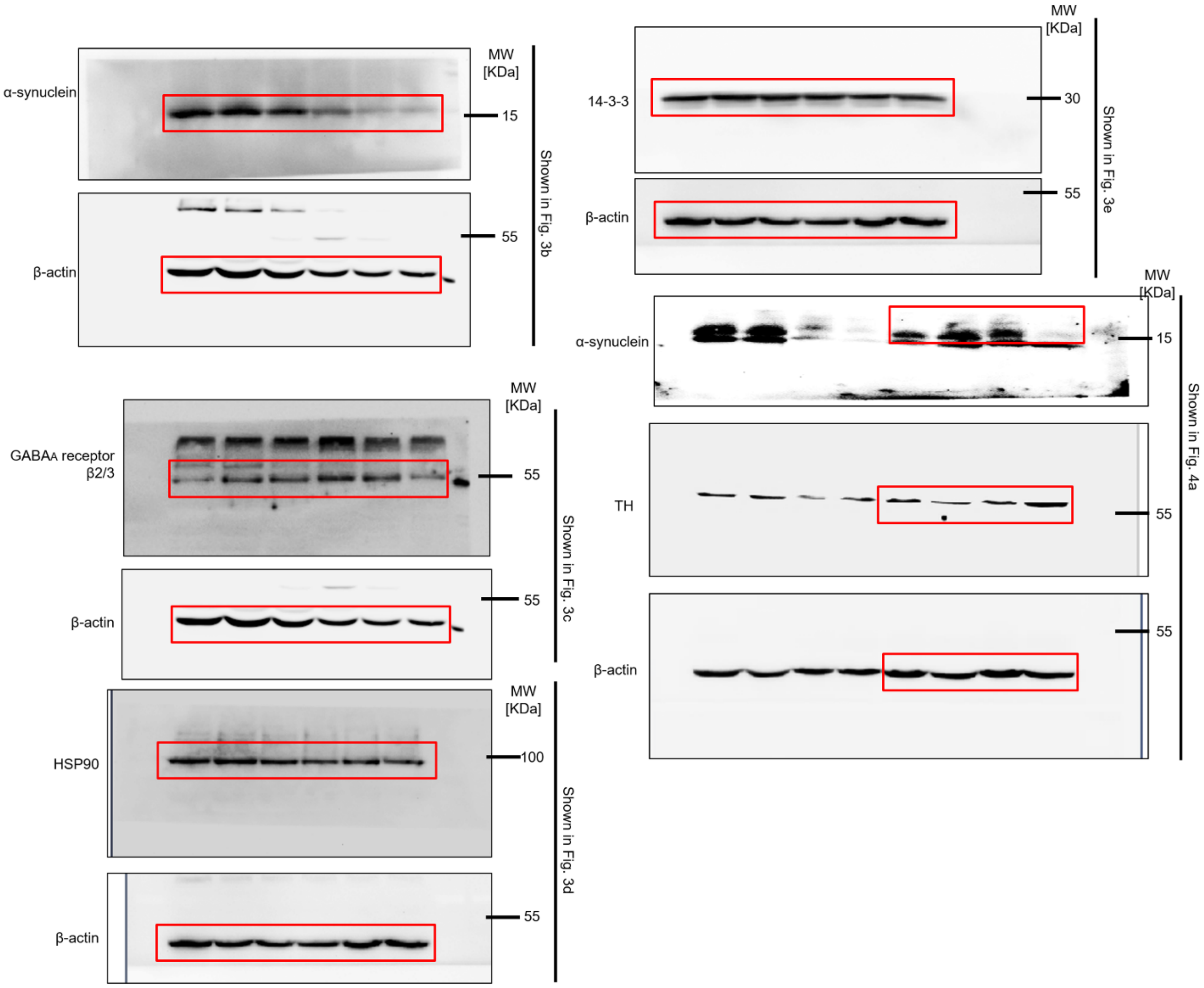

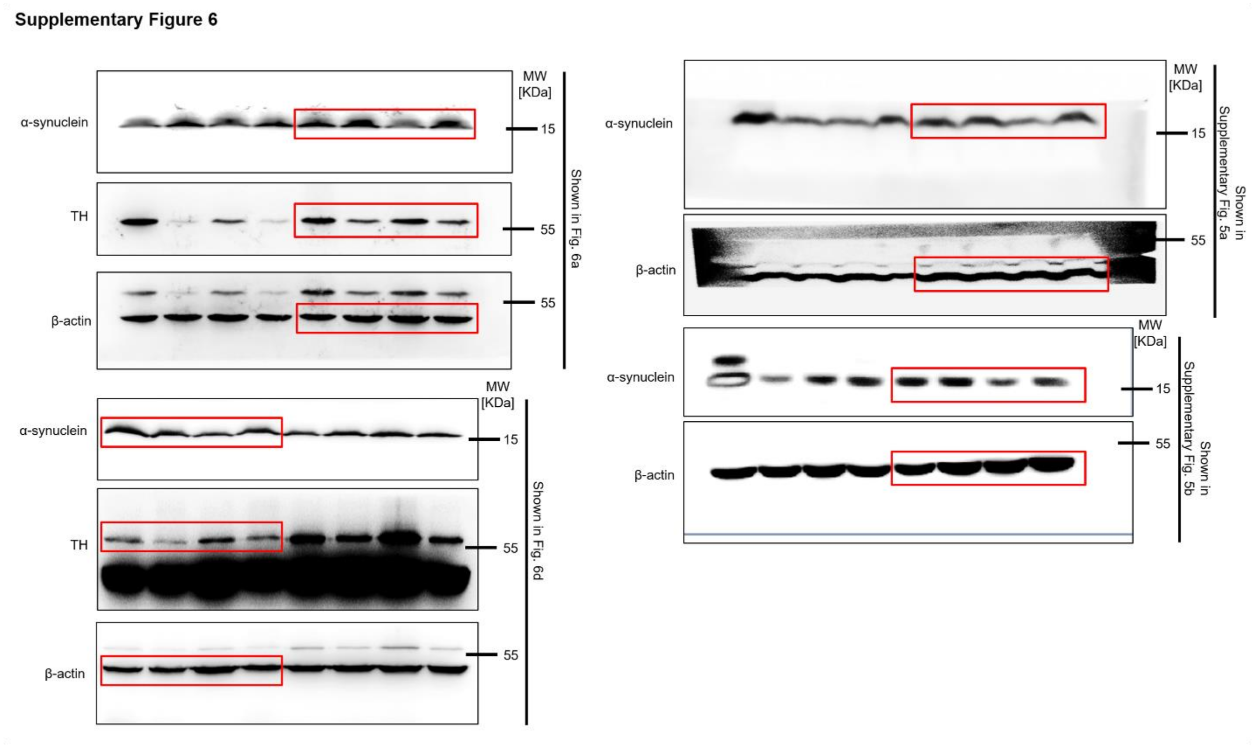

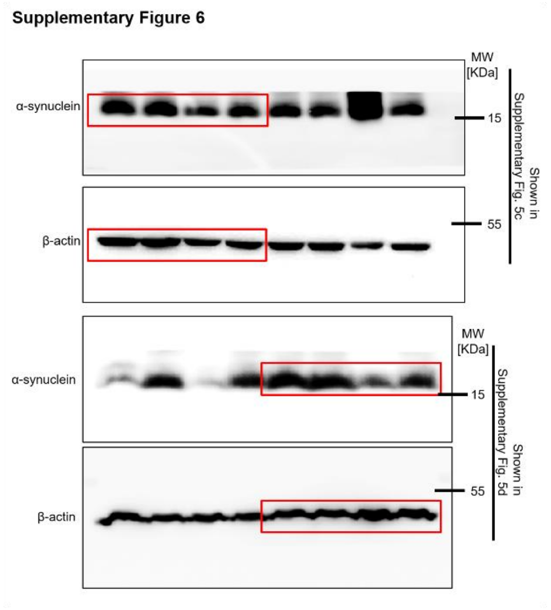

Supplementary Figure 6. Uncropped immunoblots for Fig. 1c-f, Fig. 3, Fig. 4a, Fig. 6a, Fig. 6d, and Supplementary Fig. 5.
