# Supplementary material for: Development of an α-synuclein knockdown peptide and evaluation of its efficacy in Parkinson’s disease models
Source: Commun Biol. 2021 Feb 19;4:232. doi: 10.1038/s42003-021-01746-6 (PMC7895943; doi:10.1038/s42003-021-01746-6)
Supplement: Supplementary file 3 — Description of Additional Supplementary Files [file 42003_2021_1746_MOESM3_ESM.pdf]

Description of Additional Supplementary Files

File name: Supplementary Data 1

Description: Source data for all graphs.
