# Supplementary material for: Development of an α-synuclein knockdown peptide and evaluation of its efficacy in Parkinson’s disease models
Source: Commun Biol. 2021 Feb 19;4:232. doi: 10.1038/s42003-021-01746-6 (PMC7895943; doi:10.1038/s42003-021-01746-6)
Supplement: Supplementary file 5 — Reporting Summary [file 42003_2021_1746_MOESM5_ESM.pdf]

nature research

Corresponding author(s): Yu Tian Wang

Last updated by author(s): Dec 8, 2020

Reporting Summary

Nature Research wishes to improve the reproducibility of the work that we publish. This form provides structure for consistency and transparency in reporting. For further information on Nature Research policies, see Authors & Referees and the Editorial Policy Checklist .

Statistics

For all statistical analyses, confirm that the following items are present in the figure legend, table legend, main text, or Methods section.

n/a	Confirmed	
☐	☒	The exact sample size (*n*) for each experimental group/condition, given as a discrete number and unit of measurement
☐	☒	A statement on whether measurements were taken from distinct samples or whether the same sample was measured repeatedly
☐	☒	The statistical test(s) used AND whether they are one- or two-sided<br>*Only common tests should be described solely by name; describe more complex techniques in the Methods section.*
☒	☐	A description of all covariates tested
☒	☐	A description of any assumptions or corrections, such as tests of normality and adjustment for multiple comparisons
☒	☐	A full description of the statistical parameters including central tendency (e.g. means) or other basic estimates (e.g. regression coefficient) AND variation (e.g. standard deviation) or associated estimates of uncertainty (e.g. confidence intervals)
☐	☒	For null hypothesis testing, the test statistic (e.g. *F*, *t*, *r*) with confidence intervals, effect sizes, degrees of freedom and *P* value noted<br>*Give P values as exact values whenever suitable.*
☒	☐	For Bayesian analysis, information on the choice of priors and Markov chain Monte Carlo settings
☒	☐	For hierarchical and complex designs, identification of the appropriate level for tests and full reporting of outcomes
☒	☐	Estimates of effect sizes (e.g. Cohen's *d*, Pearson's *r*), indicating how they were calculated

Our web collection on statistics for biologists contains articles on many of the points above.

Software and code

Policy information about availability of computer code

Data collection	Bio-Rad Quantity One, NIH Image J, Biacore 3000 instrument (GE Healthcare Biosciences)
Data analysis	SPSS 16.0, Matlab 2017b

For manuscripts utilizing custom algorithms or software that are central to the research but not yet described in published literature, software must be made available to editors/reviewers. We strongly encourage code deposition in a community repository (e.g. GitHub). See the Nature Research guidelines for submitting code & software for further information.

Data

Policy information about availability of data

All manuscripts must include a data availability statement. This statement should provide the following information, where applicable:

- Accession codes, unique identifiers, or web links for publicly available datasets
- A list of figures that have associated raw data
- A description of any restrictions on data availability

All source data underlying the graphs presented in the main and supplementary figures are available as Supplementary Data in Excel format. The full, uncropped blot/gel images are shown in Supplementary Fig. 6. All other data supporting the findings of this study are available within the paper and the Supplementary Information.

Field-specific reporting

Please select the one below that is the best fit for your research. If you are not sure, read the appropriate sections before making your selection.

Life sciences study design

All studies must disclose on these points even when the disclosure is negative.

Sample size	each experiment was conducted using different batches of samples for statistical analysis
Data exclusions	no data were excluded from the analyses
Replication	the experimental findings were replicated using several different batches of experimental materials. All attempts at replication were consistent
Randomization	Cell cultures and animals were randomly chosen for experiments
Blinding	not applicable

Reporting for specific materials, systems and methods

We require information from authors about some types of materials, experimental systems and methods used in many studies. Here, indicate whether each material, system or method listed is relevant to your study. If you are not sure if a list item applies to your research, read the appropriate section before selecting a response.

Materials & experimental systems

n/a	Involved in the study
☐	☒ Antibodies
☐	☒ Eukaryotic cell lines
☒	☐ Palaeontology
☐	☒ Animals and other organisms
☒	☐ Human research participants
☒	☐ Clinical data

Methods

n/a	Involved in the study
☒	☐ ChIP-seq
☒	☐ Flow cytometry
☒	☐ MRI-based neuroimaging

Antibodies

Antibodies used	Anti-α-synuclein antibody (BD Transduction Laboratories, 610786), anti-phosphorylated pS129 α-synuclein antibody (ab184674, Abcam), anti-β-actin antibody (Abcam, ab8227), anti-HA antibody (Roche, 118674231001), anti-tyrosine hydroxylase (TH) antibodies (BD Transduction Laboratories, 612300, for immunoblotting and immunohistochemistry; Novus, NB300-109, for immunocytochemistry), anti-Iba1 antibody (019-19741, Wako), anti-GABAA receptor β2/3 antibody (Millipore, 05-474), anti-HSP90 antibody (BD Transduction Laboratories, 610418), anti-14-3-3 antibody (Millipore, 06-511), normal goat serum (G9023, Sigma Aldrich), goat anti rabbit horseradish peroxidase (111-035-144, Jackson ImmunoResearch), goat anti mouse horseradish peroxidase (115-035-146, Jackson ImmunoResearch).
Validation	All these antibodies are commercial antibodies and have been widely validated by many labs in the world

Eukaryotic cell lines

Policy information about cell lines

Cell line source(s)	293 [HEK-293] (ATCC® CRL-1573™)
Authentication	As provided by ATCC
Mycoplasma contamination	Negative
Commonly misidentified lines (See ICLAC register)	Not applicable

Animals and other organisms

Policy information about studies involving animals; ARRIVE guidelines recommended for reporting animal research

Laboratory animals	Male C57BL/6 mice (20-25 g) were purchased from Charles River (Beijing Office, China); Male M83 hemizygous mice overexpressing the human A53T α-synuclein mutant (12 weeks old, 25-30 g) were purchased from the Jackson Laboratory (004479)
Wild animals	not applicable

Field-collected samples	not applicable
Ethics oversight	All experimental protocols were approved by the Chongqing Medical University Animal Care Committee and the McGill University Animal Care Committee.

Note that full information on the approval of the study protocol must also be provided in the manuscript.
